# Links between leaf anatomy and leaf mass per area of herbaceous species across slope aspects in an eastern Tibetan subalpine meadow

**DOI:** 10.1002/ece3.8973

**Published:** 2022-06-02

**Authors:** Xin’e Li, Xin Zhao, Yuki Tsujii, Yueqi Ma, Renyi Zhang, Cheng Qian, Zixi Wang, Feilong Geng, Shixuan Jin

**Affiliations:** ^1^ 38043 Division of Grassland Science College of Animal Science and Technology Yangzhou University Yangzhou Jiangsu China; ^2^ 283861 School of Natural Sciences Macquarie University Sydney New South Wales Australia; ^3^ 283861 Faculty of Science Kyushu University Fukuoka Japan; ^4^ Hawkesbury Institute for the Environment Western Sydney University Penrith New South Wales Australia; ^5^ 12426 College of Ecology Lanzhou University Lanzhou China

**Keywords:** functional trait, leaf economics spectrum, leaf morphology, leaf trait plasticity, species turnover

## Abstract

Leaf anatomy varies with abiotic factors and is an important trait for understanding plant adaptive responses to environmental conditions. Leaf mass per area (LMA) is a key morphological trait and is related to leaf performance, such as light‐saturated photosynthetic rate per leaf mass, leaf mechanical strength, and leaf lifespan. LMA is the multiplicative product of leaf thickness (LT) and leaf density (LD), both of which vary with leaf anatomy. Nevertheless, how LMA, LT, and LD covary with leaf anatomy is largely unexplored along natural environmental gradients. Slope aspect is a topographic factor that underlies variations in solar irradiation, air temperature, humidity, and soil fertility. In the present study, we examined (1) how leaf anatomy varies with different slope aspects and (2) how leaf anatomy is related to LMA, LD, and LT. Leaf anatomy was measured for 30 herbaceous species across three slope aspects (south‐, west‐, and north‐facing slopes; hereafter, SFS, WFS, and NFS, respectively) in an eastern Tibetan subalpine meadow. For 18 of the 30 species, LMA data were available from previous studies. LD was calculated as LMA divided by LT. Among the slope aspects, the dominant species on the SFS exhibited the highest LTs with the thickest spongy mesophyll layers. The thicker spongy mesophyll layer was related to a lower LD via larger intercellular airspaces. In contrast, LD was the highest on NFS among the slope aspects. LMA was not significantly different among the slope aspects because higher LTs on SFS were effectively offset by lower LDs. These results suggest that the relationships between leaf anatomy and LMA were different among the slope aspects. Mechanisms underlying the variations in leaf anatomy may include different solar radiation, air temperatures, soil water, and nutrient availabilities among the slope aspects.

## Graphical abstract

The work provides one of the most comprehensive surveys of leaf anatomical properties along a slope aspect gradient in the Tibetan subalpine meadow. By analyzing leaf anatomical properties and leaf mass per area (LMA), a key leaf functional trait, across 30 herbaceous species from three slope aspects, we revealed that different anatomical responses to micro‐environmental conditions underlie the variation in LMA among slope aspects.

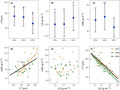

## 1 INTRODUCTION

Leaves are the main photosynthetic organs of plants and are composed of different tissues, including the cuticle, epidermis, mesophyll, and vascular systems (Evert, 2006; Fahn, 1982). The composition of each tissue within leaves (hereafter, leaf anatomical properties) is related to leaf performance, such as the capacity of photosynthesis (Terashima et al., 2011; Tholen et al., 2012), leaf mechanical strength (Choong et al., 1992; Gibson et al., 1988; Onoda et al., 2015), and evapotranspiration (Becker et al., 1986; Riederer & Schreiber, 2001; Schreiber & Riederer, 1996).

Leaf anatomical properties vary in response to environmental variables, such as solar irradiation (Chabot et al., 1979; Clements, 1905; Givnish, 1988; Hanson, 1917; Poorter et al., 2019), air temperature (Chabot & Chabot, 1977; Gratani et al., 2018; Zhou et al., 2019), precipitation (Binks et al., 2016; Cunningham et al., 1999; Fletcher et al., 2018; Turner, 1994), elevation (He et al., 2018; Körner et al., 1986; Sun et al., 2016), and soil fertility (Beadle, 1966; Cunningham et al., 1999; Tsujii et al., 2017). For example, a thick epidermis under arid environments (Körner & Kèorner, 1999; Liu et al., 2020; Sun et al., 2016) is considered an adaptive trait to protect leaf tissues from strong ultraviolet irradiation (Karabourniotis et al., 2021; Ma et al., 2012) and/or to reduce evapotranspiration from leaf surfaces (Kröber et al., 2015). A thick epidermis is also related to high mechanical strength (Onoda et al., 2015), and can result in a longer leaf lifespan (Onoda et al., 2011). A long leaf lifespan is an adaptive trait that reduces nutrient loss via leaf replacement on less fertile soils (Aerts & Chapin III, 1999; Eckstein et al., 1999; Escudero et al., 1992). Plants at high elevations (He et al., 2018; Körner et al., 1986) and in arid areas (Zhao & Huang, 1981) often have multiple mesophyll layers with high chlorophyll contents (Chen et al., 2015; Kröber et al., 2015), which may contribute to efficient light capture under high light availability. Therefore, leaf anatomical properties are an important plant trait for understanding plant adaptive responses to environmental conditions.

Leaf anatomical properties are related to leaf mass per area (LMA) (Poorter et al., 2019; Pyankov et al., 1999; de la Riva et al., 2016; Villar et al., 2013). LMA is a key morphological trait and is, in general, positively correlated with leaf lifespan and negatively correlated with light‐saturated photosynthetic rate per leaf mass and growth rate (Field & Mooney, 1986; Poorter & Van der Werf, 1998; Reich et al., 1997; Westoby et al., 2002; Wright et al., 2004). LMA can be decomposed as the product of leaf thickness (LT) and leaf density (LD) (Witkowski & Lamont, 1991). While LT is approximated to the total thickness of all tissue layers, LD depends on the composition of tissues with different densities (Niinemets, 1999; Poorter et al., 2009). The links between leaf anatomical properties and LMA have been examined in relation to LT and LD (de la Riva et al., 2016; Poorter et al., 2019; Van Arendonk & Poorter, 1994; Villar et al., 2013). For example, lower LDs are caused by larger airspaces within leaves, whereas higher LDs may be due to higher fractions of lignified cells and/or to those of mesophyll cells that have higher density than epidermis cells (Poorter et al., 2019). The results from these studies indicate that leaf anatomical properties are related to LT and LD. However, leaf anatomical properties, LMA, LT, and LD have rarely been analyzed simultaneously along natural environmental gradients (de la Riva et al., 2016).

The slope aspect provides an ideal platform to address this question. In the Northern Hemisphere, solar irradiation is the highest on equator‐facing slopes (i.e., south‐facing slopes; SFS) and the lowest on polar‐facing slopes (i.e., north‐facing slopes; NFS) (Ackerly et al., 2002; Kumar et al., 1997; Tian et al., 2001). The variation in solar radiation influences air temperature, soil humidity, and soil fertility (Li et al., 2011; Qin et al., 2021). In the present study, we investigated the leaf anatomical properties of 30 herbaceous species across three slope aspects (i.e., SFS, WFS, and NFS) in the subalpine meadow of the eastern Tibetan Plateau. LMA data for 18 of the 30 species were available from a previous study (Li et al., 2021). Using the LMA data, we examined the relationships between leaf anatomical properties and LMA, LD, or LT for the 18 species. We tested the following three hypotheses: (1) plants on SFS have higher LTs with thicker palisade mesophyll than those on other slopes because thick palisade mesophyll may contribute to efficient photosynthesis under strong solar irradiation (Lambers et al., 2008; Niinemets et al., 2005); (2) plants on SFS have higher LDs than those on other slopes because of higher fractions of palisade mesophyll layers with less intercellular airspaces and higher tissue density than epidermis layers (Poorter et al., 2019); and (3) plants on SFS have higher LMAs than other slope aspects as the result of higher LTs and LDs.

## 2 MATERIALS AND METHODS

### 2.1 Study site

**FIGURE 1:**
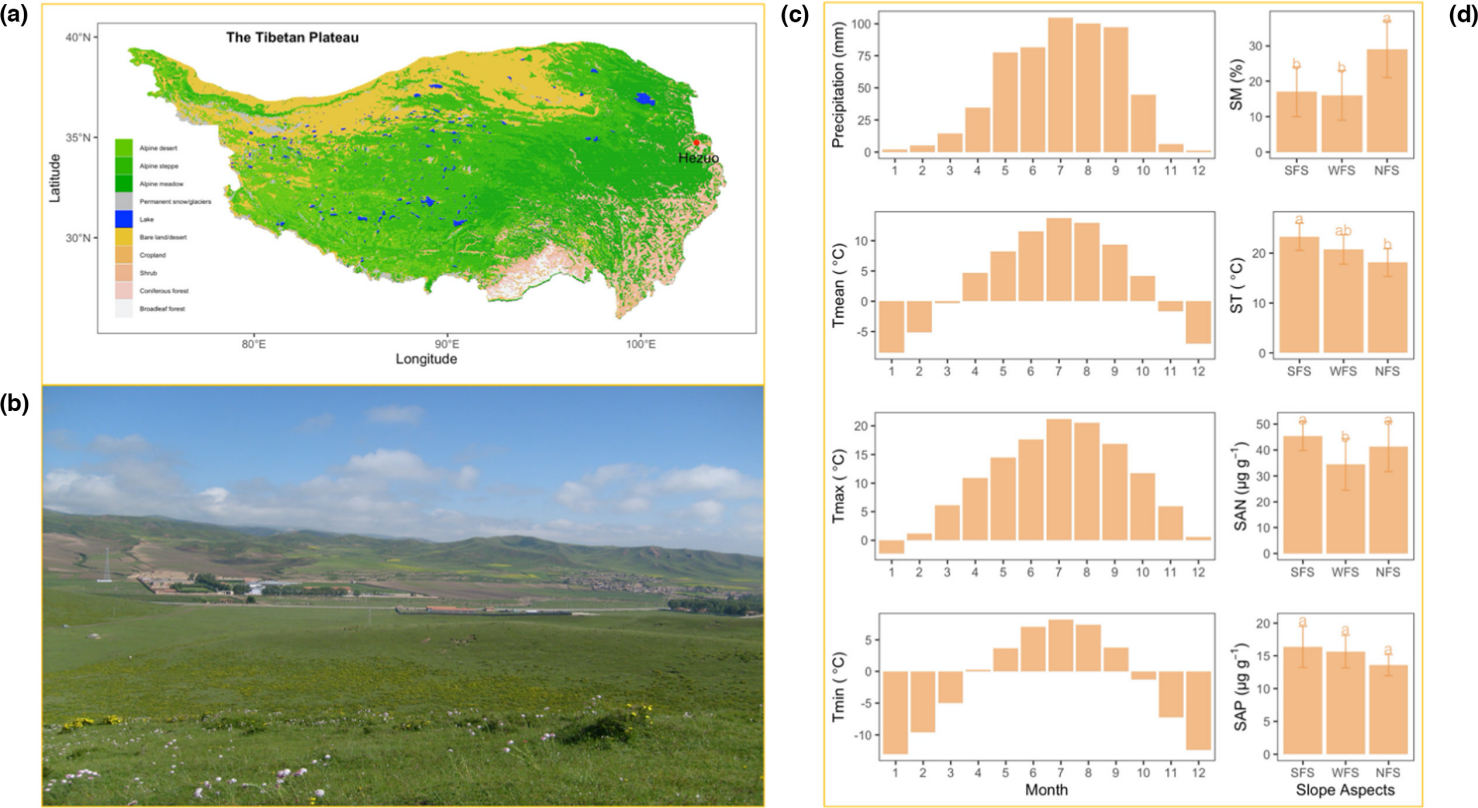
The site location on the Tibetan Plateau (a), the vegetation landscape in this region (b), and the main climate factors during the period of 1981–2017 using the climate dataset provided by National Tibetan Plateau Data Center (http://data.tpdc.ac.cn), including monthly mean annual precipitation, mean annual mean, maximum and minimum temperature (c), and description of site environments cited from Li et al. (2011, 2021) (d). SFS, WFS, and NFS represent south‐, west‐, and north‐facing slopes, respectively. Mean daily soil moisture (SM) and temperature (ST) were measured in a growing season (July–September). Soil available nitrogen (SAN) and phosphorus (SAP) (15 cm depth in topsoil) were measured by the alkali hydrolysis and the Olsen method, respectively. The error bars represent standard deviations. The one‐way ANOVA and LSD were used to test the significance

The present study was conducted in a subalpine meadow on the eastern Tibetan Plateau (34°4ʹN, 102°3ʹE, and 2960 m). The bedrock of the plateau is fluvial lacustrine clastic rock. The climate of the area reflects a humid alpine. The mean annual temperature and precipitation are 4°C and 557.8 mm, respectively (average values during 1981–2017; http://data.tpdc.ac.cn). Precipitation was concentrated during May–October. The growing season was during July–August, which is when the peaks of temperature and precipitation were recorded. Detailed location and climate data are shown in Figure 1 and can also be found in Li et al. (2011) and Li et al. (2021).

Three hills were chosen near the Research Station of Alpine Meadow and Wetland Ecosystem of Lanzhou University, Hezuo, Gansu, China. On each hill, three vegetation plots (5 m × 5 m) were established. Each of the three plots was on SFS, WFS, and NFS. The soil conditions derived from these study plots have been reported in our previous publication (Li et al., 2011, 2021). Among the slope aspects, the soil moisture on NFS was higher than those on WFS and SFS (Figure 1d). Soil temperature was the highest on SFS, followed by WFS and NFS (Figure 1d). The lowest soil available nitrogen was reported on WFS, followed by NFS and SFS, but soil available phosphorus was not significantly different among slope aspects (Figure 1d). The grassland vegetation included both graminoid and forb species. The main dominant species on SFS were graminoids. From SFS to NFS, forb species became more dominant (Li et al., 2011; Qin et al., 2016, 2019).

### 2.2 Leaf sampling

Leaves of 30 forb species were sampled during August 2018 (i.e., the peak growing season). The list of the sampled species is provided in Table S1. Species names were standardized according to the Taxonomic Name Resolution Service v. 4.1 (http://tnrs.iplantcollaborative.org/TNRSapp.html). At each plot, five mature individuals per species were sampled, kept in plastic bags, and quickly taken to the lab of the research station. Five (or three for tiny and rare species) mature, healthy, and integrated leaves were sampled from the shoot top of the plants. The sampled leaves were chemically fixed in FAA (70% ethanol: formaldehyde: glacial acetic acid = 18:1:1).

### 2.3 Leaf anatomical trait measurements

Fixed leaves were made into paraffin sections using an embedding machine (JB‐P5, WHJJ) and a slicer (RM2016, Leica). The thickness of the sliced sections was 4 µm. After deparaffinization and dehydration with a series of xylene and ethanol for approximately 1 h and staining with safranin for 1–2 h at room temperature, the sections were examined by an optical microscope, and images were taken by a microscopic camera (Nikon DS‐U3). These images were analyzed in ImageJ (NIH). The average thicknesses of the following tissue layers were measured along five vertical lines randomly drawn on the images: epidermis thickness (ET), palisade mesophyll thickness (PT), and spongy mesophyll thickness (ST). LT was also measured as the vertical thickness of leaf lamina. Three sections were examined per leaf.

### 2.4 LMA datasets

An LMA dataset measured during 2008–2010 was derived from the current nine plots and reported in our previous study (Li et al., 2021). During August 10–20, fully expanded sun leaves were sampled from 5 to 10 individuals per species at each site. The sampled leaves were scanned by a flatbed scanner (Epson, Perfection, V39, Indonesia). The scanned leaves were dried at 60°C for 2 days, and the dry mass was weighed to calculate LMA. Here, we used these LMA data because we did not repeatedly measure the LMA in the 2018 leaf sampling. In total, 50 observations including 18 species were matched between the dataset of LMA and that of leaf anatomy properties. LD was calculated as the ratio of LMA to LT. Both the LMA data and leaf anatomy data were collected in August, the peak growing season, to avoid the seasonal differences.

### 2.5 Data analysis

Linear mixed models were developed to test differences among slope aspects for leaf anatomical properties, LMA, LT, and LD, using the *lmer* function in the R package “lme4” (Bates et al.2014). In the model, “slope aspect” and “hill” were used as the fixed and random effects, respectively. “Tukey's HSD” method was used for the pairwise comparison for the leaf anatomical properties with significant differences among slope aspects using the *lsmeans* function in the R package “emmeans” (Lenth, 2021). Species means (i.e., arithmetic means by species) in each plot were used as units of replications. Correlations among LMA, LT, and LD were examined by linear mixed models with “hill” as a random factor. The same analysis was applied to the correlations of LMA, LT, or LD against thickness or the relative fraction of each tissue layer. All data were log10 transformed to normalize the data. In the correlation analyses, species means in each plot were used as units of replications.

Furthermore, we followed the procedure by Ackerly and Cornwell (2007) and Dong et al. (2020) to evaluate the contribution of intraspecific plasticity and species turnover to trait variations across the plots. The slopes in the regression of the species means against the plot‐level means were calculated for each species. These slopes are generally positive, as intraspecific variation will mirror the overall trend across the gradient but will be <1, as the trait is expected to vary less within species compared with the overall shift across plots because of intraspecific variation and species turnover (Ackerly & Cornwell, 2007). If a trait is perfectly plastic, the regression slopes derived from all species will display unity (i.e., slope = 1), while the regression line will be flat (i.e., slope = 0) if the trait shifts were all due to species turnover (Dong et al., 2020). However, slope values < 0 and >1 signify trends opposite to the community mean and indicate “overreaction,” respectively, which could also occur but are uncommon (Dong et al., 2020). The median value of all regression slopes of within‐species traits against the plot‐level mean is defined as the trait plasticity, which indicates the fraction of trait variation explained by intraspecies variation. Alternatively, the median value's complement of 1 is the measure of the fraction owing to species turnover. In our dataset, for each species sampled at three or more plots, a regression slope was obtained, and then the median species‐level slope for each trait was calculated as trait plasticity. All analyses were performed with R version 4.0.3 (R Core Team, 2020) in RStudio version 1.3.1093 (RStudio Team, 2020).

## 3 RESULTS

### 3.1 Leaf anatomical properties

**FIGURE 2:**
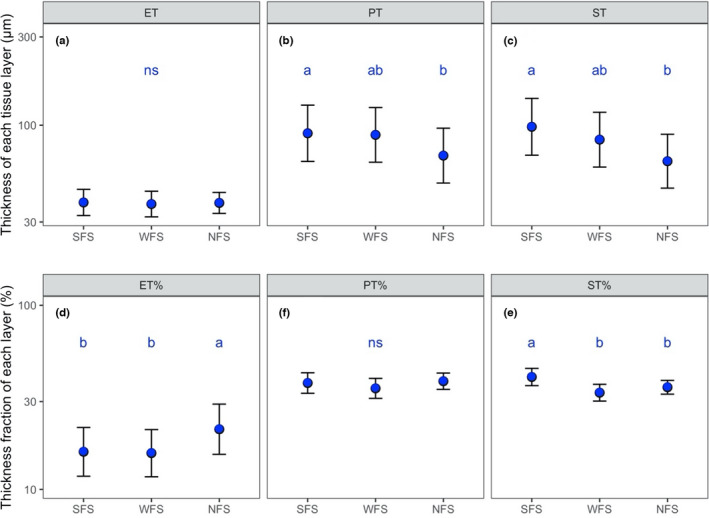
The comparison of leaf anatomical properties among different slope aspects. The comparison was conducted using the linear mixed model by treating “hill” as a random factor, and the method of Tukey's HSD was used to perform pairwise comparisons. The error bars represent standard errors. Different blue letters represent significant differences at the level of *p *< .05. “ns” represents a nonsignificant difference. SFS, WFS, and NFS represent south‐, west‐, and north‐facing slope aspects, respectively. ET, PT, and ST represent the epidermis, palisade, and spongy mesophyll thickness

Across the species, ET, ST, and PT ranged from 14.75 to 71.03, 24.23 to 395.32, and 29.83 to 222.83 µm, with relative fractions of 3.93%–44.54%, 22.97%–69.69%, and 18.59%–62.02%, respectively (Figure 2). Among the slope aspects, species on SFS exhibited significantly higher LTs than those on NFS. The higher LTs on the SFS were due to higher PTs (marginally significant with *p* = .093) and STs (Figure 2c). For relative fractions, species on SFS exhibited significantly higher ST% than those on WFS and NFS (Figure 2e). In turn, the mean ET% of the species on SFS and WFS was significantly lower than that on NFS (Figure 2d). There were no significant differences among the slope aspects for ET and PT% (Figure 2a,f).

### 3.2 Intraspecific trait plasticity

**FIGURE 3:**
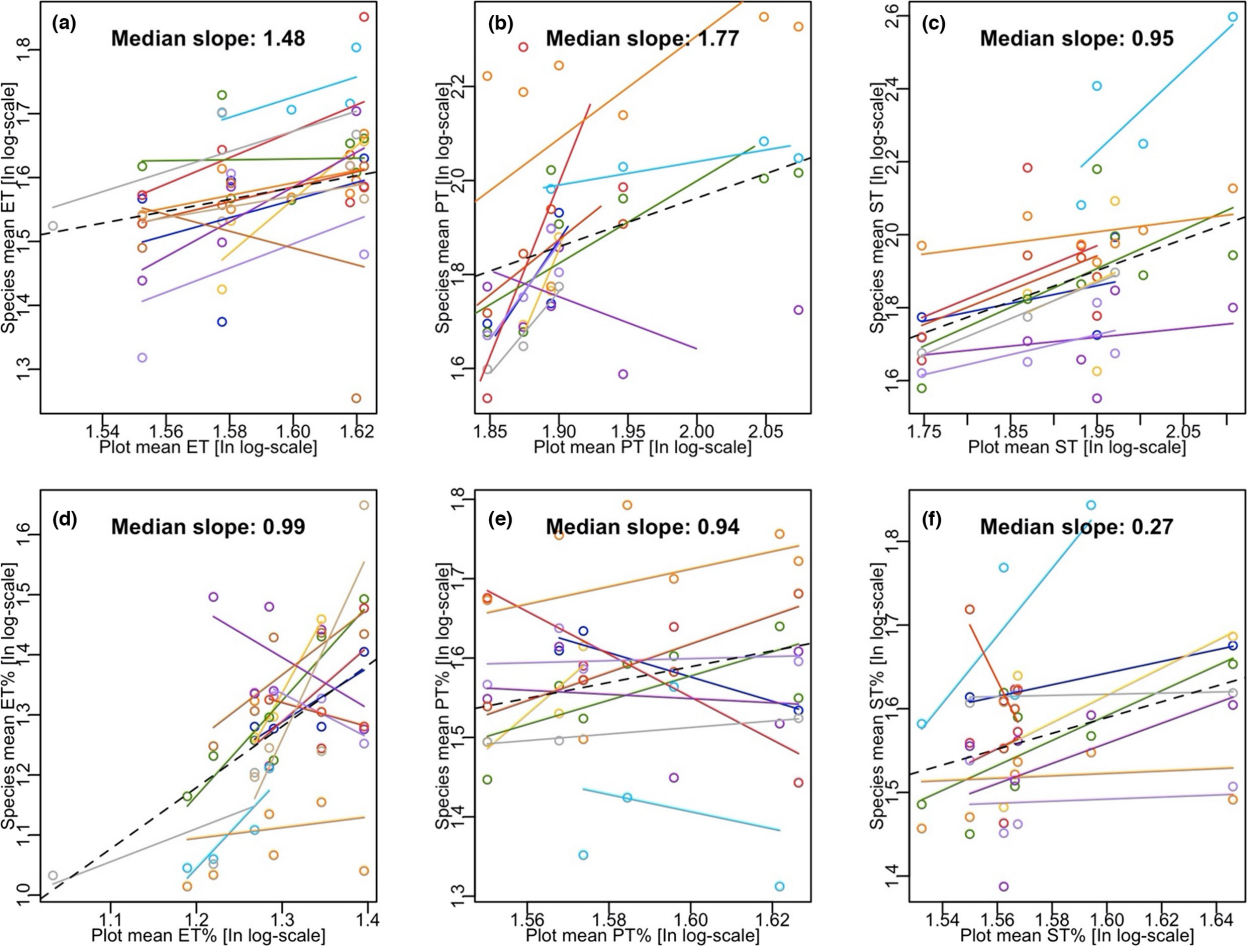
Relationships between species means and plot means for the thickness of each tissue layer (a–c) and the relative fraction of each layer (d–f). Distinct colors represent different species. The dashed black line represents the overall regression with a slope close to 1. ET, PT, and ST represent the epidermis, palisade, and spongy mesophyll thickness, respectively

The species means against the plot‐level means for each trait were plotted for distinct species (Figure 3). The median of the species‐level slope was >1 for ET and PT (1.48 and 1.77, respectively), while it was nearly equal to 1 for ST (0.95) (Figure 3a–c). Considering the relative fractions, the slope medians were nearly equal to 1 for ET% and PT% (0.99 and 0.94, respectively), except for ST% with a slope of 0.27 (Figure 3d–f).

### 3.3 The correlations of LMA with LT and LD

**FIGURE 4:**
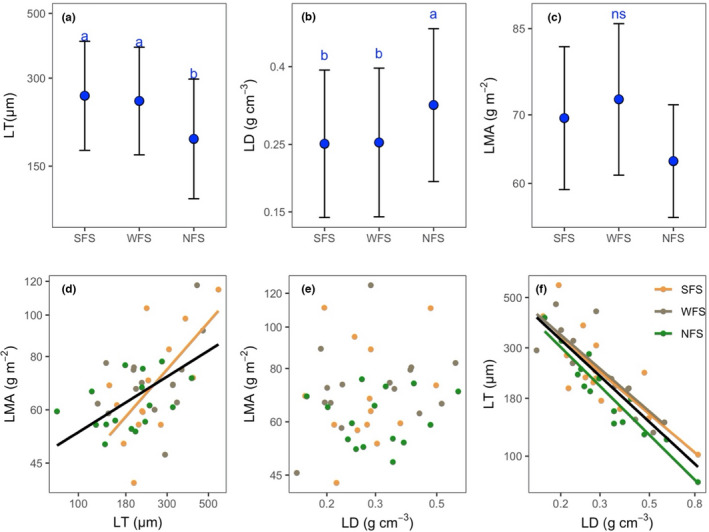
Comparison of leaf mass per area (LMA), leaf thickness (LT), and leaf density (LD) among different slope aspects (a–c) and their correlations (d–f). The comparison was conducted using the linear mixed model by treating “hill” as a random factor, and the method of Tukey's HSD was used to perform pairwise comparisons. The error bars represent standard errors. Different blue letters represent significant differences at the level of *p* < .05. The solid lines represent significant correlations at the level of *p* < .05. SFS, WFS, and NFS represent south‐, west‐, and north‐facing slope aspects, respectively. The detailed correlation information is shown in Table S2. The variables were in log scale

Among the slope aspects, species on SFS and WFS had higher LTs than those on NFS (Figure 4a), whereas LD was the highest on NFS, followed by SFS and WFS (Figure 4b). LMA was not significantly different among the slope aspects (Figure 4c).

Significant positive correlations between LMA and LT were found for the overall data and data from SFS (Figure 4d, Table S2). However, no significant correlation between LMA and LD was found across the slope aspects (Figure 4e, Table S2). Significant negative correlations between LT and LD were found across the slope aspects (Figure 4f, Table S2).

### 3.4 The correlations of LMA, LT, and LD with leaf anatomy

**FIGURE 5:**
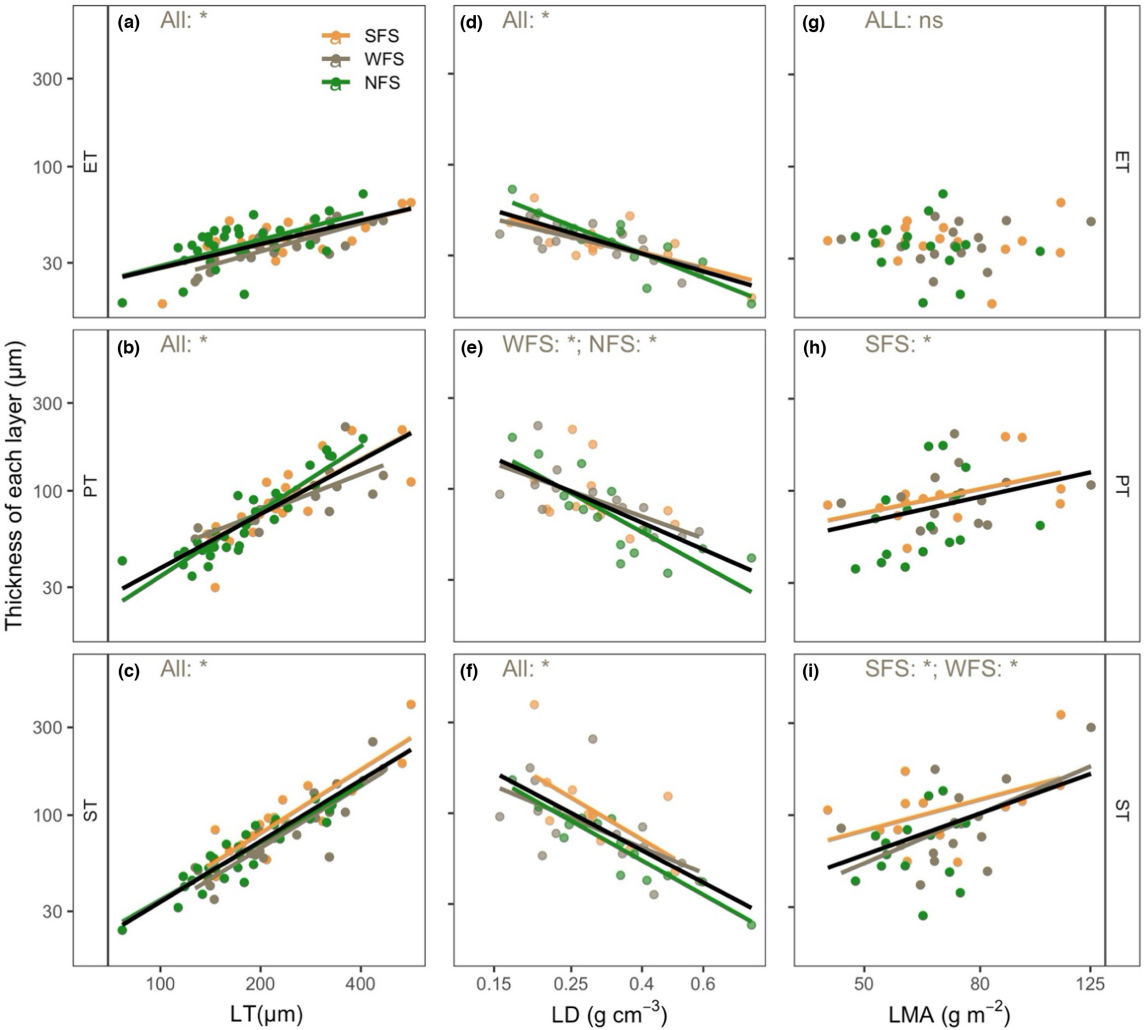
Correlations of the thickness of each tissue layer with leaf thickness (LT) (a–c), leaf density (LD) (d–f), and leaf mass per area (LMA) (h–i). The black lines are the overall regression lines. SFS, WFS, and NFS represent south‐, west‐, and north‐facing slope aspects, respectively. “All” indicates the pooled data across the three slope aspects. ET, PT, and ST represent the epidermis, palisade, and spongy mesophyll thickness, respectively. “*” and “ns” represent significant and nonsignificant, respectively. The solid lines represent significant correlations at the level of *p* < .05. The detailed correlation information is shown in Table S2

LT was significantly positively correlated with the thicknesses of all tissue layers across the slope aspects (Figure 5a–c, Table S2). Regarding LD, significant negative correlations were found against the thicknesses of all tissue layers across the slope aspects, except for PT on SFS (Figure 5d–f, Table S2). LMA was not correlated with ET but was significantly negatively correlated with PT and ST on SFS. Significant negative correlations between LMA and ST were also found on WFS and when all data were pooled (i.e., overall data) (Figure 5g–i, Table S2).

**FIGURE 6:**
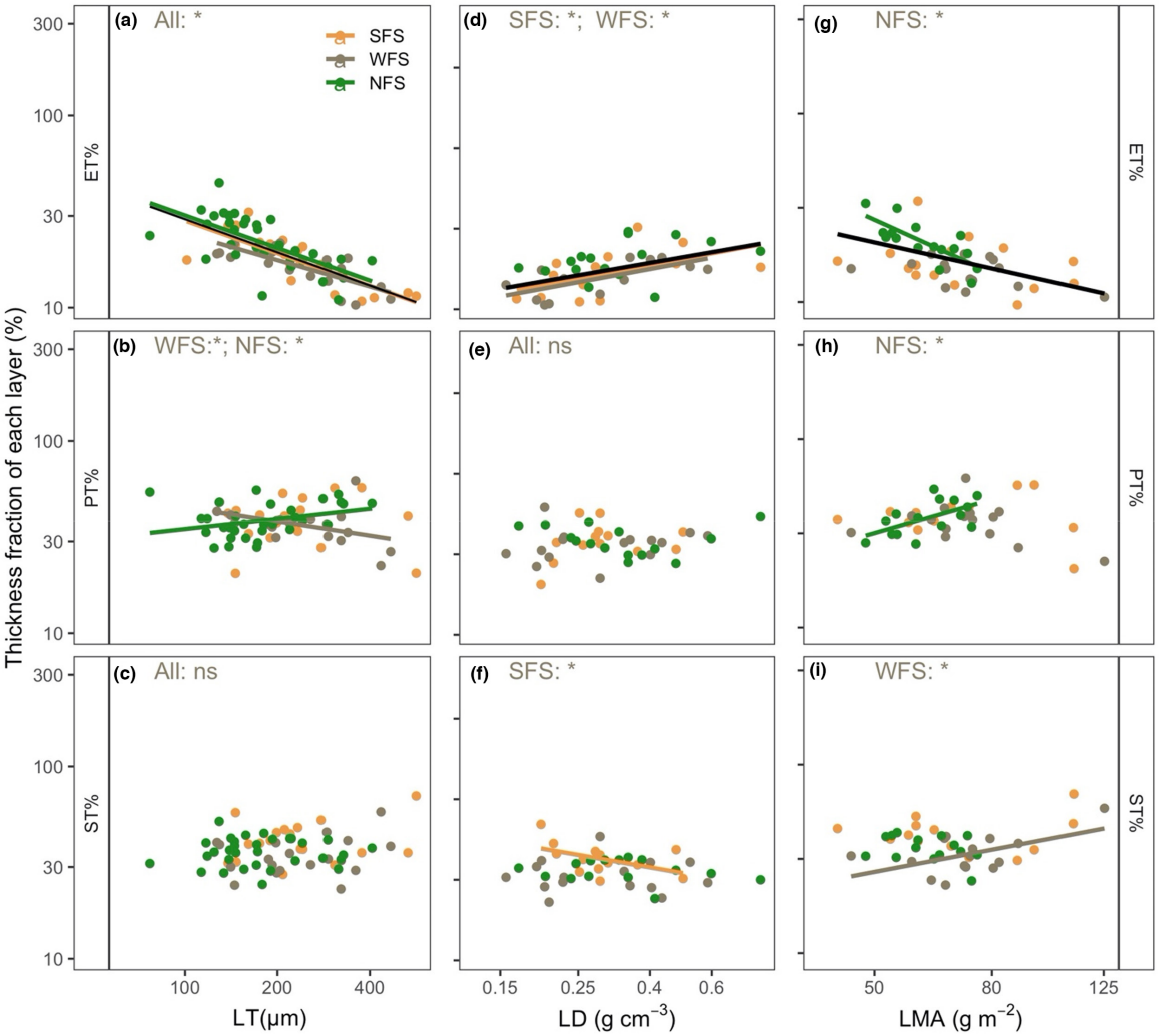
Correlations of the thickness fraction of each tissue layer with leaf thickness (LT) (a–c), leaf density (LD) (d–f), and leaf mass per area (LMA) (g–i). The black lines are the overall regression lines. SFS, WFS, and NFS represent south‐, west‐, and north‐facing slope aspects, respectively. “All” represents the pooled data across all three slope aspects. ET, PT, and ST represent the epidermis, palisade, and spongy mesophyll thickness, respectively. “*” and “ns” represent significant and nonsignificant, respectively. The solid lines represent significant correlations at the level of *p* < .05. The detailed correlation information is shown in Table S2

For the relative fraction of each tissue, LT was significantly negatively correlated with ET% (Figure 6a, Table S2) and positively correlated with PT% on NFS but negatively correlated with PT% on WFS (Figure 6b, Table S2). LD was significantly positively correlated with ET% and negatively correlated with ST% on SFS (Figure 6d,f, Table S2). LMA was negatively correlated with ET%, positively correlated with PT% on NFS, and marginally positively correlated with ST% on WFS (Figure 6g–i, Table S2).

## 4 DISCUSSION

### 4.1 Variations in leaf anatomical properties among slope aspects (Hypothesis 1)

Our first hypothesis was in part supported by our results that the dominant species on the SFS exhibited slightly thicker PTs than those on other slopes. Thick PT on SFS may contribute to efficient photosynthesis under strong solar irradiation (Lambers et al., 2008; Niinemets et al., 2005). The dominant species on SFS also exhibited higher ST and its relative fraction than on NFS. The high ST may be associated with low soil moisture on the SFS because water relation traits are known to be correlated with ST (Binks et al., 2016; Kröber et al., 2015). Spongy mesophyll offers a larger conductance pathway for lateral hydraulic flow than palisade mesophyll, which maintains turgor pressure with lower water potentials (Wylie, 1946). In contrast, the dominant species on SFS exhibited lower ET% than those on the other slope aspects because ET was not different among the slope aspects, while the LT increased on SFS.

We further examined how species turnover and intraspecific plasticity were involved in the variation in leaf anatomical properties. The median of the slopes of species means against plot means (i.e., a proxy for intraspecific trait plasticity) was >1 for ET and PT. According to Dong et al. (2020), slopes >1 (i.e., unity) indicate “overreaction.” For ST, ET%, and PT%, the median slopes were close to 1, indicating that the variations in these traits are primarily explained by intraspecific variation. In contrast, the lower plasticity of ST%, with a slope of 0.27, indicates a lower contribution of intraspecific plasticity. The plasticity of the relative fraction of each layer was on average lower than that of their thickness. This implies a relatively constant proportion of each tissue layer within species regardless of their absolute thickness.

### 4.2 Links of leaf anatomical properties with LT, LD, and LMA (hypotheses 2 and 3)

The second hypothesis was that dominant species on SFS have higher LDs with higher PT% than those on other slope aspects. However, the lowest ‐mean LD was found on the SFS. This was primarily due to the high ST% of the dominant species on the SFS. In this regard, a negative correlation was found between ST% and LD on SFS, suggesting that the low LDs on SFS were due to the large intercellular airspaces in spongy mesophyll layers. Moreover, we found that LD was positively correlated with ET%. This result is not easily explained because, in general, the epidermis has a lower density than mesophyll (Niinemets, 1999; Poorter, 2002). One possibility is that high ETs include thick cuticles, whose density is much higher than that of epidermis and mesophyll tissues (Onoda et al., 2012; Schreiber & Schönherr, 1990). The lignification of epidermal cells, small cell size, and/or higher proportions of vascular tissues and sclerenchyma may also be involved in high LD (Van Arendonk & Poorter, 1994; Castro‐Díez et al., 2000; Wang et al., 2021).

Because of the lowest LD on the SFS, our results did not support our third hypothesis that LMA would be the highest on the SFS. Nevertheless, SFS was dominated by species with higher LTs. Consequently, similar LMAs were maintained regardless of the slope aspects. More interestingly, LMA was correlated with LT but not LD across the slope aspects. This contrasts with the meta‐analysis by Poorter et al. (2009), in which LMA varied with LD rather than LT across many plant groups, including grasses, evergreen, deciduous woody species, and other plant life forms. In the present study, LT was negatively correlated with LD. This may detect an apparent trade‐off between LT and LD as a result of the convergence of LMA (=LT × LD) into a certain range.

## 5 CONCLUSION

This study is one of the most comprehensive surveys of leaf anatomical properties across slope aspects in the Tibetan meadows. Our results revealed that different anatomical mechanisms underlie the variation in LMA depending on slope aspects. Plants on SFS had higher LTs than those on NFS. The higher LTs involved higher STs, which caused relatively lower LDs on SFS than on NFS. Similar LMAs across slope aspects were maintained because higher LTs were effectively offset by lower LDs. These results suggest that the relationship between leaf anatomical properties and LMA varies with topography. Our results also showed that the relative importance of intraspecific plasticity and species turnover on anatomical variations was different among tissue types. However, it should be noted that the mechanisms underlying the variation in leaf anatomical properties remain largely unclear in part because we did not measure the heterogeneities of environmental conditions within slope aspects.

## AUTHOR CONTRIBUTIONS

Xin'e Li: Conceptualization (lead); Funding acquisition (lead); Software (lead); Visualization (lead); Writing—original draft (equal); Writing—review & editing (equal). Xin Zhao: Data curation (lead); Methodology (equal); Writing—original draft (supporting). Yuki Tsujii: Writing—original draft (equal); Writing—review & editing (equal). Yueqi Ma: Data curation (lead); Investigation (equal); Methodology (equal); Project administration (lead). Renyi Zhang: Project administration (supporting). Cheng Qian: Data curation (supporting). Zixi Wang: Data curation (supporting). Feilong Geng: Data curation (supporting). Shixuan Jin: Data curation (supporting).

## CONFLICT OF INTEREST

The authors have no relevant financial or nonfinancial interests to disclose.

## Supporting information

Supplementary Material

## Data Availability

The data that support the findings of this study have been archived in the publicly accessible repository Dryad (https://doi.org/10.5061/dryad.kd51c5b82).
