# Supplementary material for: Links between leaf anatomy and leaf mass per area of herbaceous species across slope aspects in an eastern Tibetan subalpine meadow
Source: Ecol Evol. 2022 Jun 2;12(6):e8973. doi: 10.1002/ece3.8973 (PMC9163673; doi:10.1002/ece3.8973)
Supplement: Supplementary file 1 — Supplementary Material [file ECE3-12-e8973-s001.docx]

**Supplementary Information for**

Links between leaf anatomy and leaf mass per area of herbaceous species across slope aspects in an eastern Tibetan subalpine meadow

**Xin’e Li^1, *^, Xin Zhao^1^, Yuki Tsujii^2,3,4^, Yueqi Ma^1^, Renyi Zhang^5^, Cheng Qian^1^, Zixi Wang^1^, Feilong Geng^1^, Shixuan Jin^1^**

1. Division of Grassland Science, College of Animal Science and Technology, Yangzhou University, Yangzhou, 225009, Jiangsu, China.
2. School of Natural Sciences, Macquarie University, Sydney, NSW, Australia.
3. Faculty of Science, Kyushu University, 744, Motooka, Nishi-ku, Fukuoka, 819-0395 Japan
4. Hawkesbury Institute for the Environment, Western Sydney University, Penrith, NSW, Australia.
5. Department of Life Sciences, Lanzhou University, Lanzhou, 730000, Gansu, China.

*Corresponding author

Division of Grassland Science, College of Animal Science and Technology, Yangzhou University

No.48 Wenhui Road, Yangzhou, Jiangsu, China

Email: [lixine@yzu.edu.cn](about:blank)

This file includes:

Table S1

Table S2

Table S1 The average value of each species measured in this region. ET, PT, and ST represent the epidermis, palisade, spongy mesophyll thickness, respectively. SFS, WFS, NFS represent south-, west-, north-facing slope aspects, respectively.

| **Species** | **ET (µm)** | **PT (µm)** | **ST (µm)** | **LT (µm)** | **SFS** | **WFS** | **NFS** |
| --- | --- | --- | --- | --- | --- | --- | --- |
| *Ajania przewalskii* | 14.748 |  |  | 425.307 | 1 |  |  |
| *Anaphalis lactea* | 30.452 | 47.836 | 61.911 | 149.305 | 1 |  | 1 |
| *Artemisia desertorum* | 43.447 | 102.151 | 82.184 | 352.367 | 1 | 1 |  |
| *Artemisia tangca* | 36.213 | 44.629 | 32.124 | 113.039 |  |  | 1 |
| *Bupleurum* | 27.821 | 46.370 | 68.022 | 137.849 | 1 | 1 |  |
| *Dracocephalum tanguticum* | 46.595 | 111.035 | 56.786 | 208.475 | 1 |  |  |
| *Euphrasia pectinata* | 38.440 | 71.005 | 83.411 | 192.239 |  |  | 1 |
| *Gentiana macrophylla* | 54.162 | 108.838 | 237.217 | 440.056 | 1 | 1 | 1 |
| *Gueldenstaedtia verna* | 38.837 | 161.272 | 102.120 | 309.374 | 1 | 1 | 1 |
| *Heteropappus hispidus* | 35.646 | 63.237 | 70.321 | 186.294 | 1 | 1 | 1 |
| *Lagedium sibiricum* | 35.869 | 74.556 | 90.720 | 186.434 |  | 1 | 1 |
| *Leontopodium leontopodioides* | 35.317 | 61.079 | 78.201 | 177.290 | 1 | 1 | 1 |
| *Medicago falcata* | 38.023 | 54.328 | 52.963 | 151.161 | 1 | 1 | 1 |
| *Nepeta cataria* | 42.880 | 50.186 | 60.208 | 172.586 |  |  | 1 |
| *Oxytropis* | 47.194 | 100.605 | 87.687 | 239.317 |  | 1 | 1 |
| *Plantago asiatica* | 44.976 | 107.758 | 92.444 | 267.243 | 1 | 1 |  |
| *Polygonum viviparum* | 36.819 | 72.415 | 75.797 | 202.463 |  | 1 | 1 |
| *Potentilla anserina* | 19.211 | 50.274 | 33.421 | 127.720 |  | 1 |  |
| *Potentilla bifurca* | 31.969 | 68.735 | 61.350 | 187.260 |  |  | 1 |
| *Potentilla fragarioides* | 33.081 | 61.481 | 49.729 | 144.216 |  | 1 |  |
| *Potentilla multifida* | 37.753 | 69.129 | 60.181 | 192.472 | 1 | 1 | 1 |
| *Potentilla supina* | 36.891 | 58.070 | 43.038 | 138.153 |  | 1 |  |
| *Silene sp* | 23.293 |  |  | 229.046 | 1 |  |  |
| *Sanguisorba officinalis* | 54.666 | 89.340 | 55.072 | 190.365 |  |  | 1 |
| *Saussurea* | 34.607 | 66.540 |  |  |  |  | 1 |
| *Saussurea brunneopilosa* | 60.152 | 185.002 | 148.736 | 427.155 | 1 |  | 1 |
| *Silene gallica* | 43.231 | 100.458 | 91.887 | 230.222 |  | 1 | 1 |
| *Stachys sieboldii* | 44.726 | 49.459 | 55.691 | 145.183 |  |  | 1 |
| *Stellera chamaejasme* | 42.868 | 82.486 | 84.752 | 226.812 | 1 | 1 | 1 |
| *Taraxacum mongolicum* | 38.565 | 78.555 | 120.520 | 249.802 | 1 |  |  |

Table S2 Correlations among leaf thickness (LT), leaf density (LD), leaf mass per area (LMA), and leaf anatomical properties across and within slope aspects. The linear mixed model was used to detect the correlations by treating “site” as the random factor. The bold font represents significant correlations at the level of P < 0.05. ET = epidermis thickness, ST = spongy tissue thickness, PT = palisade tissue thickness, and LT = leaf thickness. “lower. CT” and “upper. CT” means the lower and upper bounds of the confidence interval for the regression slopes. SFS, WFS, NFS represent south-, west- and north-facing slope aspects, respectively.

| **Correlations of anatomical properties with LT, LD and LMA** | | | | | | | | | | | | | | | | | | |
| --- | --- | --- | --- | --- | --- | --- | --- | --- | --- | --- | --- | --- | --- | --- | --- | --- | --- | --- |
|  |  | **R^2^** | **P** | **slope** | **lower.**  **CL** | **upper.**  **CL** |  | **R^2^** | **P** | **slope** | **lower.CL** | **upper.CL** |  | **R^2^** | **P** | **slope** | **lower.CL** | **upper.CL** |
| ET-LT | SFS | **0.48** | **<0.001** | **0.42** | **0.21** | **0.63** | ET-LD | **0.51** | **0.003** | **-0.48** | **-0.77** | **-0.20** | ET-LMA | <0.001 | 0.940 | -0.02 | -0.62 | 0.58 |
|  | WFS | **0.67** | **<0.001** | **0.51** | **0.33** | **0.68** |  | **056** | **0.001** | **-0.45** | **-0.69** | **-0.21** |  | 0.07 | 0.331 | 0.29 | -0.33 | 0.92 |
|  | NFS | **0.39** | **<0.001** | **0.47** | **0.26** | **0.68** |  | **0.76** | **<0.001** | **-0.75** | **-0.99** | **-0.51** |  | **0.03** | **0.469** | **-0.33** | **-1.28** | **0.62** |
|  | Overall | **0.43** | **<0.001** | **0.42** | **0.31** | **0.54** |  | **0.59** | **<0.001** | **-0.55** | **-0.69** | **-0.42** |  | <0.001 | 0.938 | 0.14 | -0.35 | 0.37 |
| ST-LT | SFS | **0.79** | **<0.001** | **1.14** | **0.85** | **1.43** | ST-LD | **0.40** | **0.021** | **-1.13** | **-2.05** | **-0.21** | ST-LMA | **0.24** | **0.087** | **0.82** | **-0.14** | **1.79** |
|  | WFS | **0.77** | **<0.001** | **1.12** | **0.80** | **1.43** |  | **0.37** | **0.016** | **-0.80** | **-1.41** | **-0.18** |  | **0.32** | **0.029** | **1.32** | **0.16** | **2.49** |
|  | NFS | **0.86** | **<0.001** | **1.04** | **0.88** | **1.19** |  | **0.87** | **<0.001** | **-1.07** | **-1.32** | **-0.82** |  | 0.04 | 0.450 | 0.65 | -1.16 | 2.46 |
|  | Overall | **0.81** | **<0.001** | **1.10** | **0.97** | **1.23** |  | **0.52** | **<0.001** | **-1.01** | **-1.31** | **-0.70** |  | **0.24** | **0.001** | **1.10** | **0.48** | **1.72** |
| PT-LT | SFS | **0.63** | **<0.001** | **0.98** | **0.59** | **1.36** | PT-LD | 0.14 | 0.203 | -0.52 | -1.36 | 0.32 | PT-LMA | **0.24** | **0.089** | **0.64** | **-0.12** | **1.39** |
|  | WFS | **0.63** | **<0.001** | **0.70** | **0.43** | **0.97** |  | **0.55** | **0.001** | **-0.68** | **-1.05** | **-0.32** |  | 0.03 | 0.550 | 0.25 | -0.64 | 1.14 |
|  | NFS | **0.84** | **<0.001** | **1.17** | **0.99** | **1.36** |  | **0.65** | **<0.001** | **-1.05** | **-1.50** | **-0.59** |  | 0.11 | 0.210 | 0.91 | -0.57 | 2.39 |
|  | Overall | **0.73** | **<0.001** | **0.97** | **0.83** | **1.11** |  | **0.49** | **<0.001** | **-0.83** | **-1.10** | **-0.57** |  | **0.14** | **0.013** | **0.68** | **0.15** | **1.22** |
| ET%-LT | SFS | **0.63** | **<0.001** | **-0.56** | **-0.77** | **-0.36** | ET%-LD | **0.28** | **0.042** | **0.40** | **0.02** | **0.78** | ET%-LMA | 0.11 | 0.228 | -0.37 | -0.99 | 0.26 |
|  | WFS | **0.58** | **<0.001** | **-0.46** | **-0.65** | **-0.26** |  | **0.48** | **0.004** | **0.40** | **0.15** | **0.65** |  | 0.193 | 0.101 | -0.34 | -0.76 | 0.08 |
|  | NFS | **0.43** | **<0.001** | **-0.58** | **-0.79** | **-0.33** |  | 0.14 | 0.151 | 0.25 | -0.10 | 0.60 |  | **0.60** | **<0.001** | **-1.38** | **-2.03** | **-0.73** |
|  | Overall | **0.56** | **<0.001** | **-0.57** | **-0.69** | **-0.45** |  | **0.27** | **<0.001** | **0.38** | **0.19** | **0.56** |  | **0.28** | **<0.001** | **-0.68** | **-1.01** | **-0.35** |
| ST%-LT | SFS | 0.05 | 0.345 | 0.13 | -0.16 | 0.43 | ST%-LD | **0.24** | **0.088** | **-0.38** | **-0.83** | **0.07** | ST%-LMA | 0.01 | 0.725 | 0.08 | -0.41 | 0.58 |
|  | WFS | 0.07 | 0286 | 0.15 | -0.13 | 0.42 |  | <0.001 | 0.980 | 0.00 | -0.35 | 0.34 |  | **0.29** | **0.039** | **0.58** | **0.04** | **1.12** |
|  | NFS | <0.001 | 0.869 | 0.01 | -0.15 | 0.18 |  | 0.05 | 0.409 | -0.09 | -0.31 | 0.14 |  | 0.10 | 0.257 | -0.35 | -1.00 | 0.29 |
|  | Overall | 0.03 | 0.158 | 0.09 | -0.03 | 0.21 |  | 0.03 | 0.247 | -0.11 | -0.30 | 0.08 |  | 0.028 | 0.280 | 0.167 | -0.141 | 0.476 |
| PT%-LT | SFS | <0.001 | 0.969 | -0.01 | -0.38 | **0.37** | PT%-LD | 0.09 | 0.333 | 0.28 | -0.33 | 0.89 | PT%-LMA | <0.001 | 0.858 | -0.05 | -0.67 | 0.57 |
|  | WFS | **0.204** | **0.052** | **-0.24** | **-0.49** | **0.002** |  | 0.04 | 0.493 | 0.11 | -0.23 | 0.44 |  | 0.14 | 0.166 | -0.40 | -0.98 | 0.19 |
|  | NFS | **0.109** | **0.064** | **0.18** | **-0.01** | **0.36** |  | 0.02 | 0.623 | -0.07 | -0.37 | 0.23 |  | **0.34** | **0.022** | **0.88** | **0.15** | **1.60** |
|  | Overall | <0.001 | 0.879 | -0.01 | -0.15 | 0.13 |  | 0.01 | 0.481 | 0.07 | -0.13 | 0.27 |  | 0.004 | 0.675 | -0.068 | -0.391 | 0.256 |
